# High prevalence of geriatric syndromes in older adults

**DOI:** 10.1371/journal.pone.0233857

**Published:** 2020-06-05

**Authors:** Angela M. Sanford, John E. Morley, Marla Berg-Weger, Janice Lundy, Milta O. Little, Kathleen Leonard, Theodore K. Malmstrom

**Affiliations:** 1 Division of Geriatric Medicine, Saint Louis University School of Medicine, St. Louis, MO, United States of America; 2 School of Social Work, Saint Louis University, St. Louis, MO, United States of America; 3 Department of Social Work and Geriatric Care Management, Perry County Memorial Hospital, Perryville, MO, United States of America; 4 Division of Geriatric Medicine, Duke University School of Medicine, Durham, NC, United States of America; 5 Department of Psychiatry and Behavioral Neuroscience, Saint Louis University School of Medicine, St. Louis, MO, United States of America; Universita degli Studi di Napoli Federico II, ITALY

## Abstract

**Introduction:**

The geriatric syndromes of frailty, sarcopenia, weight loss, and dementia are highly prevalent in elderly individuals across all care continuums. Despite their deleterious impact on quality of life, disability, and mortality in older adults, they are frequently under-recognized. At Saint Louis University, the Rapid Geriatric Assessment (RGA) was developed as a brief screening tool to identify these four geriatric syndromes.

**Materials and methods:**

From 2015–2019, the RGA, comprised of the FRAIL, SARC-F, Simplified Nutritional Appetite Questionnaire (SNAQ), and Rapid Cognitive Screen (RCS) tools and a question on Advance Directives, was administered to 11,344 individuals ≥ 65 years of age across Missouri in community, office-based, hospital, Programs of All-Inclusive Care for the Elderly (PACE), and nursing home care settings. Standard statistical methods were used to calculate the prevalence of frailty, sarcopenia, weight loss, and dementia across the sample.

**Results:**

Among the 11,344 individuals screened by the RGA, 41.0% and 30.4% met the screening criteria for pre-frailty and frailty respectively, 42.9% met the screening criteria for sarcopenia, 29.3% were anorectic and at risk for weight loss, and 28.1% screened positive for dementia. The prevalence of frailty, risk for weight loss, sarcopenia, and dementia increased with age and decreased when hospitalized patients and those in the PACE program or nursing home were excluded.

**Conclusions:**

Using the RGA as a valid screening tool, the prevalence of one or more of the geriatric syndromes of frailty, sarcopenia, weight loss, and dementia in older adults across all care continuums is quite high. Management approaches exist for each of these syndromes that can improve outcomes. It is suggested that the brief RGA screening tool be administered to persons 65 and older yearly as part of the Medicare Annual Wellness Visit.

## Introduction

**Fig 1:**
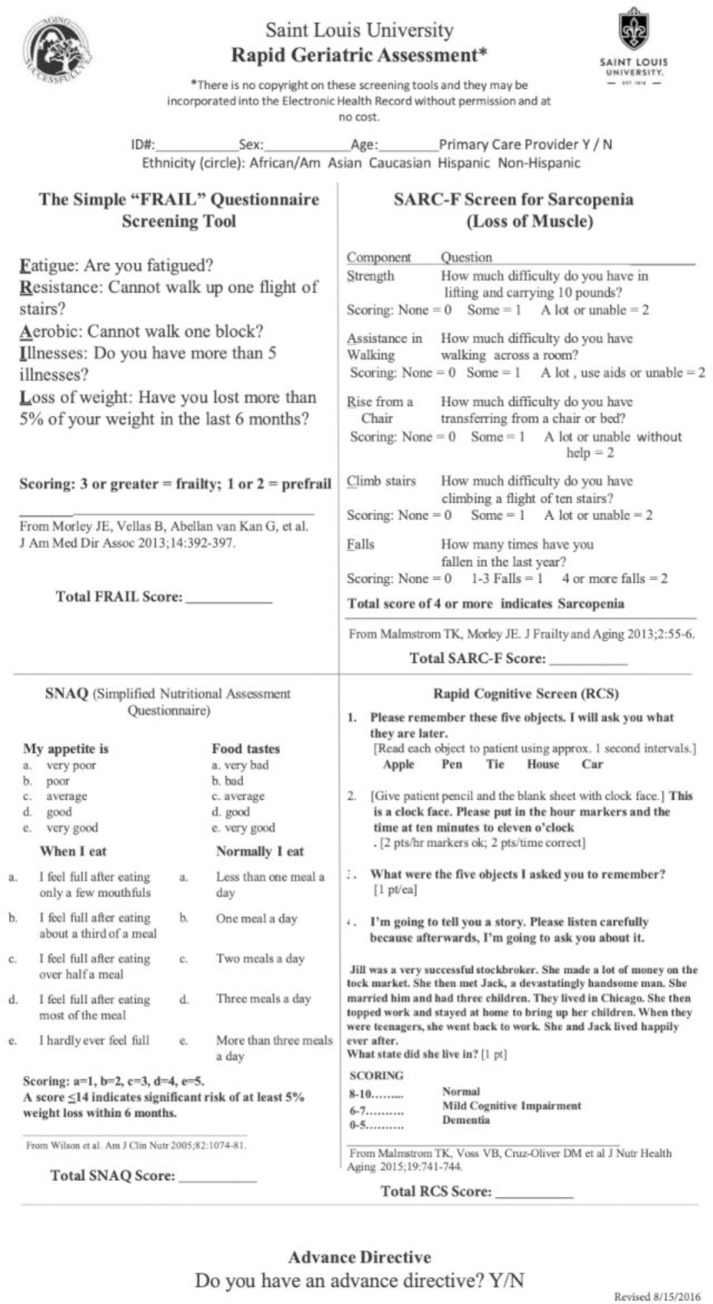
Rapid Geriatric Assessment (RGA). Screening tool developed at Saint Louis University in 2015 to identify four different geriatric syndromes; frailty, sarcopenia, weight loss, and dementia.

The geriatric syndromes of frailty, sarcopenia, weight loss, and dementia are highly prevalent in older adults across all care continuums. [1–4] Despite deleterious impacts on quality of life, disability, and mortality in older adults, geriatric syndromes are frequently under-recognized.^4^ This may be in part due to the limited time the typical primary care physician has to interact with each patient,[5] which is not adequate for comprehensive evaluation of all of the complex needs of a geriatric patient. At Saint Louis University, the Rapid Geriatric Assessment (RGA) was developed as a brief screening tool to identify these four geriatric syndromes.[6] (Fig 1) The RGA, which takes 4–5 minutes to complete, was originally developed in 2015 to encourage screening in the geriatric population despite the time constraints faced in healthcare settings.

The RGA consists of four separate validated screening tools; the Rapid Cognitive Screen (RCS) to screen for dementia,[7] the Simplified Nutritional Appetite Questionnaire (SNAQ) to screen for weight loss,[8,9] the FRAIL scale to screen for frailty,[10] and the SARC-F to screen for sarcopenia,[11,12] as well as one yes/no question as to whether or not the individual has an advanced directive. These identified geriatric syndromes were selected to be part of the comprehensive screen because of their significant negative impact on the quality of life of older adults and because there are several potential underlying treatable conditions that may contribute to each syndrome. Additionally, the presence of any one or more of these geriatric syndromes can prohibit successful aging in place and be a predictor of poor health outcomes.

The goal for screening is to identify treatable geriatric syndromes in order to implement multi-faceted, targeted interventions, utilizing the interprofessional team before further functional impairment or morbidity from these geriatric syndromes occurs. The RGA can be completed on paper or an electronic device and can be implemented in an electronic medical record system. It also fulfills the screening component of the Medicare Annual Wellness Exam.[13] Additionally, it can be administered by office staff or other members of the interprofessional team, enhancing its practicality in a busy office or hospital setting. The aim of this study was to assess the prevalence of the four geriatric syndromes of dementia, frailty, sarcopenia, and weight loss utilizing the RGA across care continuums in Missouri. It was hypothesized that these syndromes were common in those greater than 65 years of age and had increased prevalence with age. The recognition and awareness of geriatric syndromes in older persons will improve patient-centered care and markedly improve healthcare outcomes.

## Materials and methods

From July 2015 through June 2019, the RGA, comprised of the FRAIL, SARC-F, SNAQ, and RCS screening tools and a question on Advance Directives, was administered to 11,344 individuals 65 years and older across Missouri. Persons younger than 65 years of age (n = 933) were excluded from this report. These screenings were conducted by members of the interdisciplinary team, such as physicians, physicians’ assistants, nurse practitioners, medical assistants, nurses, social workers, physical therapists, occupational therapists, and speech therapists in a variety of settings across the healthcare continuum, i.e., community (n = 2688), office-based medical practice (n = 6445), hospital (n = 1268), Programs of All-Inclusive Care for the Elderly (PACE) (n = 31), and nursing home (n = 912) care settings. Screenings were undertaken in all 6 congressional districts in Missouri and occurred in both urban and rural areas. Not all four components of the RGA were completed in each individual and were accounted for as “missing variables” in the result section.

The data collection was part of the Geriatrics Workforce Enhancement Program (GWEP), funded by the Health Resources and Services Administration (HRSA). All data was anonymized and collected as part of the reporting requirements for HRSA. The Saint Louis University Institutional Review Board approved data collection and analysis. Chi-square tests were done to compare RGA results by age group (65–74, 75–84, 85+), and site (physician medical practice, community screening, nursing home, PACE, and hospital), to compare advance directive (yes/no) by age group and site, and to compare the overlap between frailty (as determined by the FRAIL scale) and sarcopenia (as determined by the SARC-F).

## Results

**Fig 2:**
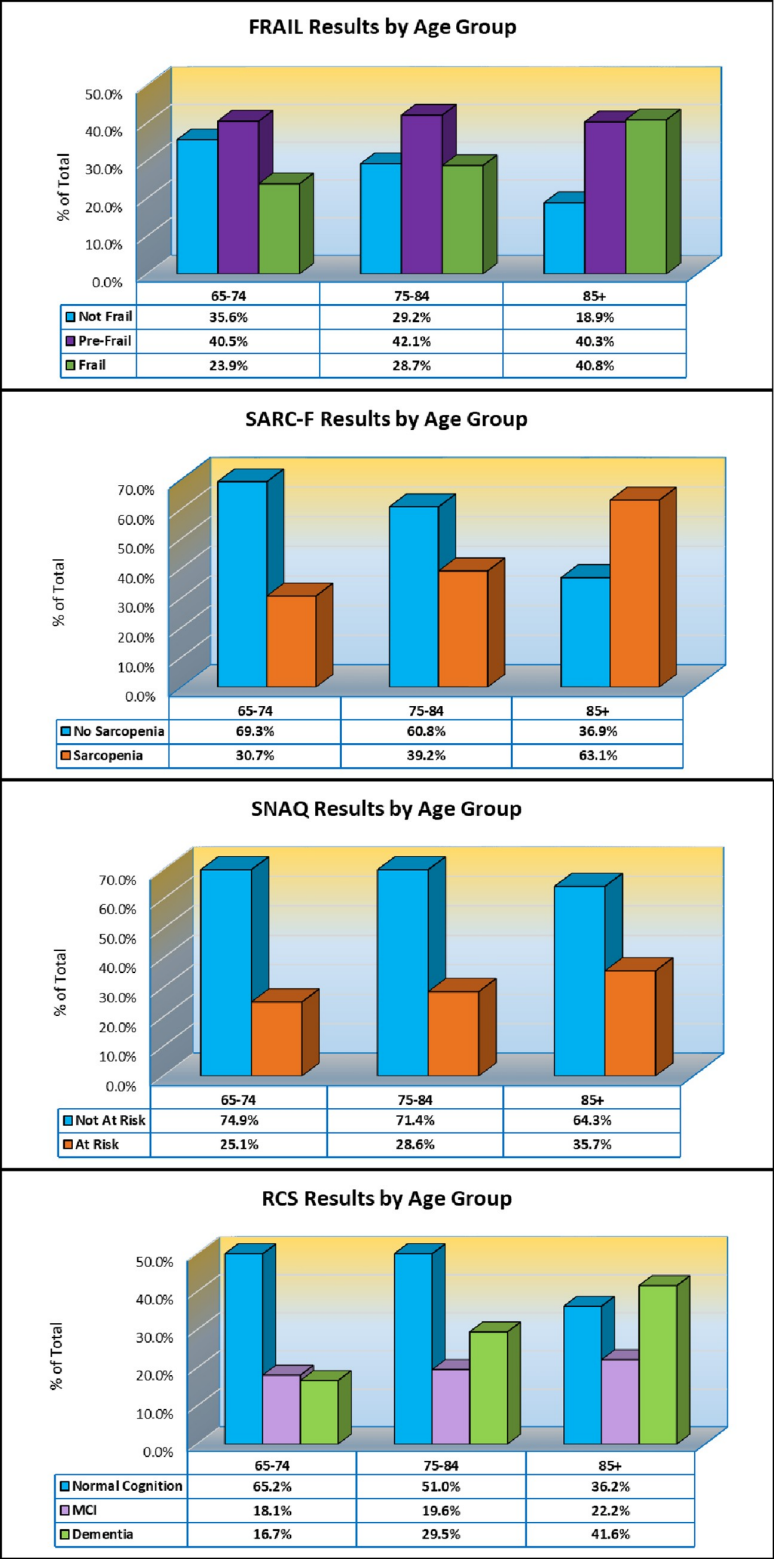
Rapid Geriatric Assessment (RGA) by age group. FRAIL, SARC-F, Simplified Assessment Questionnaire (SNAQ), and Rapid Cognitive Screen (RCS) results by age group (ages 65–74, 75–84, and 85+).

The RGA results for the FRAIL, SARC-F, SNAQ, and RCS screening tools are shown by age group distribution in Fig 2. Across all age groups, the FRAIL scale identified 30.4% of persons who screened positive for frailty (n = 3354/11042, n = 302 missing) with frailty rates increasing with age: 23.9% screening positive ages 65–74 (n = 947; n = 98 missing), 28.7% screening positive ages 75–84 (n = 1149; n = 121 missing), and 40.8% screening positive ages 85+ (n = 1258; n = 83 missing)(p < .001). Sarcopenia on the SARC-F was observed in 42.9% of persons (n = 4723/11018, n = 326 missing). Across age groups 65–74, 75–84, and 85+, sarcopenia prevalence increased from 30.7% (n = 1204) to 39.2% (n = 1574) to 63.1% (n = 1945), respectively (p < .001). Risk for weight loss according to the SNAQ was 29.3% across all age groups (n = 3192/10884, n = 460 missing), and also increased with age. 25.1% of those ages 65–74 screened positive for weight loss (n = 977), 28.6% of those ages 75–84 screened positive (n = 1133), and 35.7% ages 85+ (n = 1082) screened positive for weight loss (p < .001). On the RCS, 28.1% of individuals across all age groups met the screening criteria for dementia (n = 2645/9419, n = 1925 missing) and 19.7% met the screening criteria for mild cognitive impairment (n = 1859/9419, n = 1925 missing). Dementia and MCI, respectively, were highest among those aged 85+ (41.6%, n = 1054; 22.2%, n = 561) followed by those ages 75–84 (29.5%, n = 1019; 19.6%, n = 677) and then those ages 65–74 (16.7%, n = 572; 18.1%, n = 621) (p < .001).

**Fig 3:**
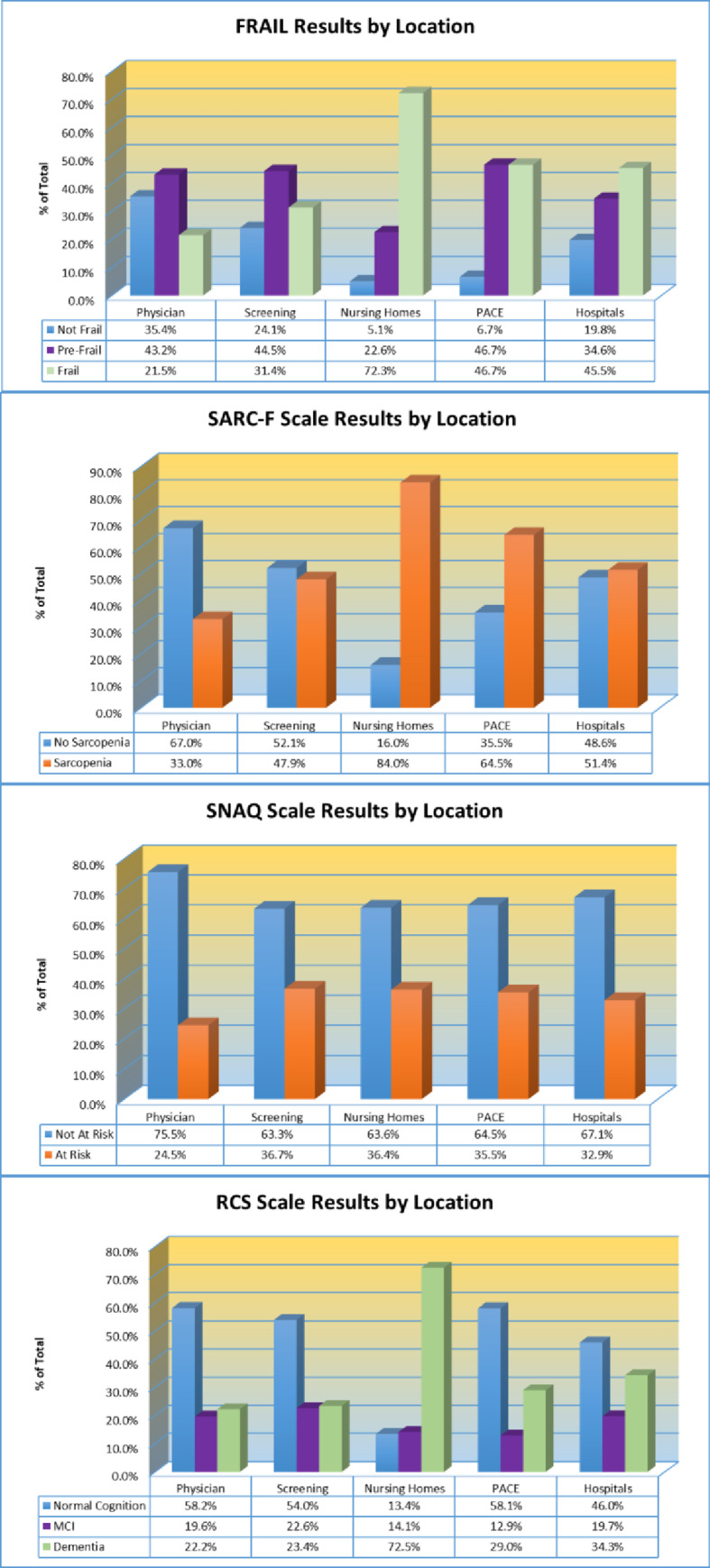
Rapid Geriatric Assessment (RGA) by location. FRAIL, SARC-F, Simplified Assessment Questionnaire (SNAQ), and Rapid Cognitive Screen (RCS) results by location where screening occurred. Locations included physician office, community screening, nursing home, Programs of All-Inclusive Care for the Elderly (PACE), or hospital.

**Fig 4:**
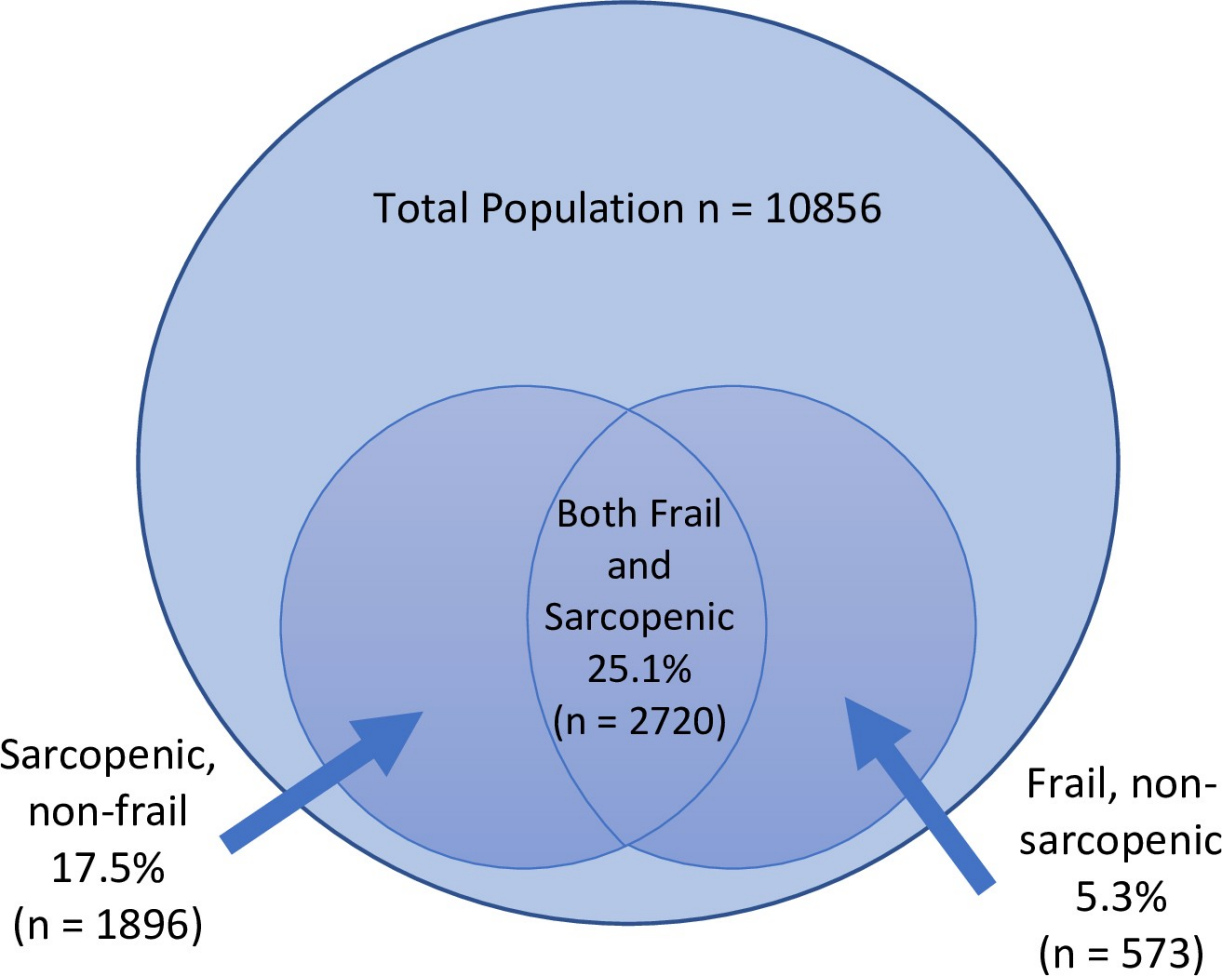
Overlap of frailty (FRAIL) and Sarcopenia (SARC-F). Most individuals who screened positive for frailty or sarcopenia concurrently screened positive for both geriatric syndromes, creating a large overlap between the two syndromes.

Fig 3 provides the results of the RGA by care setting, including hospitals (n = 1268, 11.2%), nursing homes (n = 912, 8.0%), PACE (n = 31, 0.3%), physician offices (n = 6445, 56.8%), and community screenings (n = 2688, 23.7%). Significant differences in the prevalence rates of frailty, sarcopenia, risk for weight loss, and dementia across locations were identified (all p_s_ < .001). The FRAIL, SARC-F, and RCS had the highest percentage of positive screenings in nursing homes: FRAIL (72.3%, n = 601/831, n = 81 missing), SARC-F (84.0%, n = 758/902, n = 10 missing), and RCS (72.5%, n = 565/779, n = 133 missing). Both the hospital and the PACE program locations had higher positive screens than physician office or community screening in the following areas: FRAIL (hospitals: 45.5%, n = 546/1199, n = 69 missing and PACE: 46.7%, n = 14/30, n = 1 missing), SARC-F (hospitals: 51.4%, n = 616/1198, n = 70 missing and PACE: 64.5%, n = 20/31), and RCS (hospitals: 34.3%, n = 384/1121, n = 147 missing and PACE: 29.0%, n = 9/31). All groups had smaller, but similar positives for the SNAQ (hospitals: 32.9%, n = 384/1166, n = 102 missing; nursing homes: 36.4%, n = 299/821, n = 91 missing; PACE: 35.5%, n = 11/31; physician offices: 24.5%, 1521/6203, n = 242 missing; community screenings: 36.7%, n = 977/2663, n = 25 missing) with the lowest rate observed in the physician offices group. A significant overlap (25.1%, n = 2720/10856, n = 488 missing; p < .001) between persons with frailty on the FRAIL (30.4%, n = 3293/10856) and SARC-F positives (42.5%, n = 4616/10856) was identified. Of the total study population, only 5.3% were frail and not sarcopenic, and only 17.5% were sarcopenic and not frail (Fig 4).

**Fig 5:**
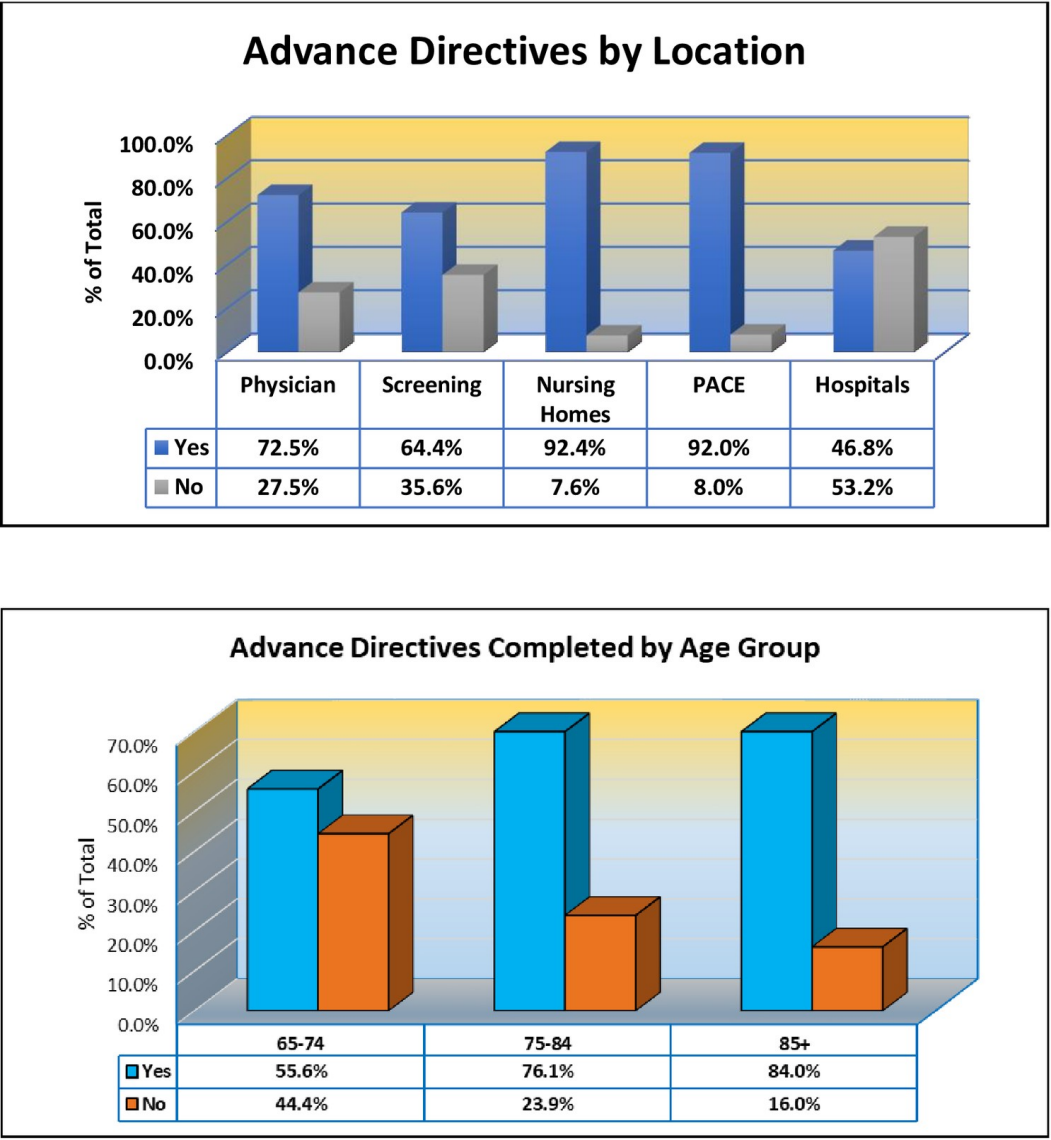
Advance directives completed by location and age group. Number of individuals who have completed advance directives by age group (ages 65–74, 75–84, and 85+) and location (locations include physician office, community screening, nursing home, Programs of All-Inclusive Care for the Elderly (PACE), or hospital).

The highest number of persons having completed advance directives (n = 5766/11344; n = 5578 missing) were in nursing homes (92.4%, n = 671/726, n = 186 missing) and PACE (92.0%, n = 23/25, n = 6 missing), while the lowest number completed were in hospitalized patients (46.8%, n = 139/297, n = 971 missing) (Fig 5). Overall, the age group who had completed the most advance directives were those aged 85 and over (84.0%, n = 1461/1740, n = 1423 missing), while the smallest number of advance directives had been completed by persons ages 65 to 74 years (55.6%, n = 1067/1918, n = 2140 missing) (p < .001; Fig 5).

## Discussion

Our study reveals that the geriatric syndromes of sarcopenia, frailty, dementia, and weight loss are highly prevalent across the care continuum in Missouri elders. Despite a negative impact on quality of life and increased morbidity in the elderly, as well as an inverse relationship with successful aging in place, geriatric syndromes are not routinely screened for or identified in most healthcare settings.[14] Highlighting this point is a report released in 2019 by the Alzheimer’s Association which concluded that while 94% of primary care physicians consider it important to assess all patients >65 years of age for cognitive impairment, one example of a geriatric syndrome, only 16% of seniors receive routine cognitive screening by these same primary care physicians.[15] Lack of screening results in a large percentage of the population who may have unrecognized cognitive impairment. One reason for inadequate screening of geriatric syndromes is the time required to administer screening tests. The RGA, developed as a brief screening tool requiring 4–5 minutes for administration, can be administered by any member of the interprofessional team and in any care setting—outpatient, hospital, and nursing home. It should be noted that while any healthcare team member can administer the screening, a physician, physician’s assistant, and/or nurse practitioner should interpret and discuss the results with the patient. The components of the RGA all have been validated in multiple countries and settings.[16–20]

A second reason that routine screening does not typically occur is the perception that no disease-modifying treatments exist for the identified geriatric syndrome. On the contrary, there are, in fact, multi-faceted interventions, often simple in nature, that can be implemented to halt or slow the progression of the geriatric syndrome or even reverse its deleterious effects. For example, reducing polypharmacy, treating sleep apnea, or improving depression symptoms can help improve cognitive impairment and also reduce fatigue, which is a component of frailty. Treatable causes of anorexia and weight loss have been identified and exercise programs have been shown to reduce sarcopenia.[21] Screening is imperative so that healthcare professionals are aware of limitations, for example, in those who are cognitively impaired, that may prohibit patients from taking their medications or following instructions correctly. It is important to recognize cognitive impairment early in the disease course to implement preventative measures before crises such as driving dangerously or living alone unsafely occurs. Earlier recognition also allows the individual to participate in shared decision-making regarding end-of-life care preferences and financial and legal decisions before their memory loss progresses to a point where they are no longer able to make sound decisions. Our study revealed that across all age groups, only 71.7% of geriatric persons had an advance directive, indicating much of the population is at risk for undergoing unwanted interventions at the end-of-life.

A third reason that screening for geriatric syndromes is not routinely implemented is that there are no large-scale screening recommendations by impactful medical organizations for geriatric syndromes. For example, in 2014, the United States Preventative Services Task Force (USPSTF), an organization that publishes many of our recognized screening guidelines, concluded that the current evidence was insufficient to assess the balance of benefits and harms of screening for cognitive impairment in older adults.[22] This is despite the fact that there are many older adults who cannot follow simple instructions, who are driving unsafely, and who are at risk for financial scams because of their unidentified cognitive impairment. Equally as unfortunate, there is no mention of other geriatric syndromes, such as frailty, sarcopenia, or weight loss in their screening guidelines. Screening is often the first step to making a diagnosis and having diagnosis in place frequently initiates treatment. Unfortunately, at the present time, we are left with consensus guidelines and case finding approaches.[23]

Our prevalence study highlights the need for routine screening for geriatric syndromes in those >65 years of age due to the high frequency of these syndromes across the population and geriatric care continuum. Although few in number, other population-level studies have yielded similar results.[24,25] Bulut et al, found the frequency of dementia to be 21.6%, sarcopenia to be 31.7%, and frailty to be 28.3% in a sample of 2,816 community-dwelling Turkish elders.[26] These result were lower than that found in our study, however our study included all care settings and was likely comprised of more functionally and cognitively-impaired individuals, including more individuals living in poor socio-economic conditions. Another smaller study conducted in Sweden found frailty in 38% and pre-frailty in 28.3% of the study population, which included both inpatient and outpatient older adults >65 years of age. Frailty was more common in the hospitalized patients, while pre-frailty was more prevalent in the outpatient population.[27] We do not know for certain how the prevalence of these syndromes has changed or will change over time, but another study conducted in Swedish elders found the prevalence of geriatric syndromes to be stable over time.[28] Of note, our study found the overlap between frailty and sarcopenia risk to be quite high with 25.1% of older adults screening positive for both frailty and sarcopenia. Of those screening positive for risk of either frailty or sarcopenia, only 5.3% were at risk for frailty alone and only 17.5% were at risk for sarcopenia alone. Liguori et al., have published an intriguing article looking at the complex relationship between frailty and sarcopenia and have identified in-depth therapeutic strategies to improve health outcomes in older adults who are at risk of becoming frail and/or sarcopenic, further supporting the need for screening and identification of these syndromes.[29]

Our study had several notable weaknesses. First, the sample population reflected older individuals across Missouri. While individuals from rural, suburban, and urban areas were included, our sample may not be representative of other population samples. Many participants had incomplete variables and did not complete all four screening tests included in the Rapid Geriatric Assessment, leading to missing data which may have impacted our results. Additionally, the screenings were conducted by many different members of the healthcare team who, while trained to administer the RGA, had variable education, training, and experience levels precluding inter-rater reliability assessment.

The strengths in our study include the large sample size of 11,344 participants aged 65 and older, and the administration across the geriatric care continuum (included nursing home residents, hospitalized patients, as well as older adults seen in outpatient clinic settings and at community screening events). While this study only evaluated prevalence, in the future, it will be beneficial to evaluate the effectiveness of targeted interventions and explore other strategies aimed to reduce the prevalence and frequency of geriatric syndromes. Additionally, there may be a role for the prospective validation of our screening instrument in our specific older adult population as was done in Italian older adults by Abete, et al.[30]

## Conclusions

The RGA, a valid screening tool, identified the prevalence of one or more of the geriatric syndromes of frailty, sarcopenia, weight loss, and dementia in the older adult population across all care continuums to be high. Despite the ill effects on quality of life, functional status, and mortality, these syndromes are frequently under-recognized and under-treated. The purpose of screening for geriatric syndromes is to enable the implementation of multi-faceted, individualized interventions to prevent further morbidity and disability and promote successful aging in place.

## Supporting information

S1 Data (XLS)
